# Supplementary figures and images for: Morphological traits: predictable responses to macrohabitats across a 300 km scale
Source: PeerJ. 2014 Mar 4;2:e271. doi: 10.7717/peerj.271 (PMC3961160; doi:10.7717/peerj.271)

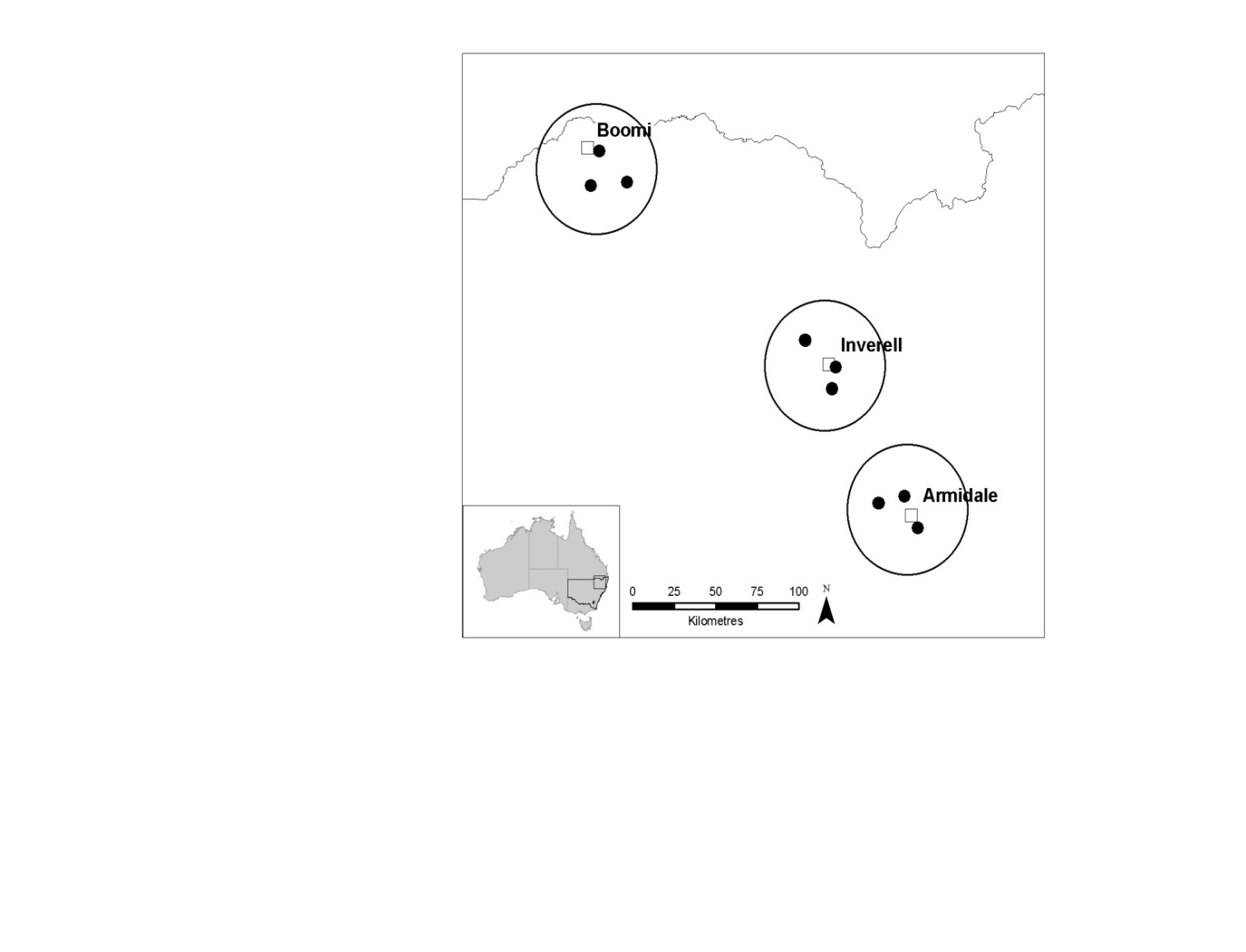

Supplement: Figure S1 — Circle outlines encompass the three individual zones while solid circles represent the three paired sites within each zone. Squares represent the main town of the zone where the following climatic data was collect from (Average climatic data for the past 15 years and elevation for Boomi, Inverell and Armidale: Mean minimum temperature (°C): Boomi = 12.9, Inverell = 7.4, Armidale = 7.1; Mean maximum temperature (°C): 27.6, 23.9, 20.3; Average rainfall (mm/yr): 505.9, 805.3, 791.5; Elevation (m.a.s.l.) = 160, 582, 980) (BOM 2010). [file peerj-02-271-s001.docx]
