# Supplementary material for: Morphological traits: predictable responses to macrohabitats across a 300 km scale
Source: PeerJ. 2014 Mar 4;2:e271. doi: 10.7717/peerj.271 (PMC3961160; doi:10.7717/peerj.271)
Supplement: Table S1 [file peerj-02-271-s002.docx]

| TRAITS | Weber’s | MII | EW | EL | HL | HW | ML | TT | SL | AL | MSL | MHL | FEM | TIB | TAR | MP | LP | ST | SP | T |
| --- | --- | --- | --- | --- | --- | --- | --- | --- | --- | --- | --- | --- | --- | --- | --- | --- | --- | --- | --- | --- |
| *Continuous measures* |  |  |  |  |  |  |  |  |  |  |  |  |  |  |  |  |  |  |  |  |
| Weber’s Length | 1.00 | 0.06 | -0.34 | -0.18 |  |  | 0.30 | -0.11 | -0.39 |  | -0.11 | -0.18 | 0.13 | 0.18 | 0.05 | 0.09 | 0.15 | -0.15 | 0.00 |  |
| Min. inter-eye distance (MII) | 0.06 | 1.00 | 0.40 | 0.30 | -0.11 | 0.17 | -0.12 | -0.08 | 0.09 | 0.08 | 0.39 | 0.16 | 0.35 | 0.28 | 0.37 | 0.22 | 0.07 | 0.39 | 0.00 |  |
| Eye width (EW) | -0.34 | 0.40 | 1.00 |  | 0.39 | 0.50 | 0.35 | 0.23 | 0.33 | 0.49 | 0.29 | 0.21 | 0.27 | 0.30 | 0.31 |  |  | 0.36 | 0.00 |  |
| Eye length (EL) | -0.18 | 0.30 | **0.96** | 1.00 | 0.28 | 0.36 | 0.50 | 0.28 | 0.33 |  | 0.19 | 0.12 | 0.37 | 0.43 | 0.40 |  |  | 0.26 | 0.00 |  |
| Head length (HL) | **-0.74** | -0.11 | 0.39 | 0.28 | 1.00 |  | -0.08 | 0.20 | 0.44 |  | 0.00 | 0.20 | -0.03 | -0.01 | 0.10 | 0.02 | -0.13 | 0.02 | 0.00 |  |
| Head width (HW) | **-0.67** | **0.17** | 0.50 | 0.36 | **0.85** | 1.00 | 0.11 | 0.28 | 0.34 |  | 0.25 |  | 0.05 | 0.02 | 0.13 | -0.03 | -0.26 | 0.27 | 0.00 |  |
| Mandible length (ML) | 0.30 | -0.12 | **0.35** | **0.50** | -0.08 | 0.11 | 1.00 |  | -0.17 | 0.04 | -0.04 | 0.17 | 0.00 | 0.07 | -0.03 | 0.16 | 0.12 | -0.02 | 0.00 |  |
| Top tooth (TT) | -0.11 | -0.08 | 0.23 | 0.28 | 0.20 | 0.28 | 0.75 | 1.00 | -0.12 | 0.14 | -0.10 | 0.03 | -0.27 | -0.23 | -0.22 | -0.11 | -0.19 | -0.11 | 0.00 |  |
| Scape length (SL) | -0.39 | 0.09 | 0.33 | 0.33 | 0.44 | **0.34** | -0.17 | -0.12 | 1.00 |  | -0.17 | 0.13 |  |  |  |  | 0.39 | -0.05 | 0.00 |  |
| Antenna length (AL) | -0.53 | 0.08 | **0.49** | 0.50 | **0.59** | **0.53** | 0.04 | 0.14 | **0.92** | 1.00 | -0.18 | 0.10 | 0.54 |  |  | 0.46 | 0.34 | -0.06 | 0.00 |  |
| Max. spine length (MSL) | -0.11 | **0.39** | 0.29 | 0.19 | 0.00 | 0.25 | -0.04 | -0.10 | -0.17 | -0.18 | 1.00 | 0.42 | -0.28 | -0.28 | -0.30 | -0.07 | -0.20 |  | 0.00 |  |
| Max. hair length^a^ (MHL) | -0.18 | **0.16** | 0.21 | 0.12 | 0.20 | 0.52 | 0.17 | 0.03 | 0.13 | 0.10 | 0.42 | 1.00 | -0.08 | -0.16 | -0.11 | -0.23 | -0.37 | 0.49 | 0.00 |  |
| Mid-femur length (FEM) | 0.13 | 0.35 | 0.27 | 0.37 | -0.03 | 0.05 | 0.00 | -0.27 | **0.61** | **0.54** | -0.28 | -0.08 | 1.00 |  |  |  |  | -0.26 | 0.00 |  |
| Mid-tibia length (TIB) | 0.18 | 0.28 | 0.30 | 0.43 | -0.01 | 0.02 | 0.07 | -0.23 | **0.60** | **0.54** | -0.28 | -0.16 | **0.98** | 1.00 |  |  |  | -0.26 | 0.00 |  |
| Mid-tarsus length (TAR) | 0.05 | 0.37 | **0.31** | **0.40** | 0.10 | 0.13 | -0.03 | -0.22 | **0.68** | **0.64** | -0.30 | -0.11 | **0.98** | **0.98** | 1.00 |  |  | -0.27 | 0.00 |  |
| *Count measures* |  |  |  |  |  |  |  |  |  |  |  |  |  |  |  |  |  |  |  |  |
| Maxillary palp segments (MP) | 0.09 | 0.22 | **0.65** | **0.73** | 0.02 | -0.03 | 0.16 | -0.11 | 0.52 | 0.46 | -0.07 | -0.23 | **0.68** | **0.75** | **0.69** | 1.00 |  | -0.03 | 0.00 |  |
| Labial palp segments (LP) | 0.15 | **0.07** | **0.55** | **0.65** | -0.13 | -0.26 | 0.12 | -0.19 | 0.39 | 0.34 | -0.20 | -0.37 | 0.59 | **0.67** | 0.58 | **0.94** | 1.00 | -0.15 | 0.00 |  |
| Spines (alitrunk) (ST) | -0.15 | **0.39** | 0.36 | 0.26 | 0.02 | 0.27 | -0.02 | -0.11 | -0.05 | -0.06 | **0.98** | **0.49** | -0.26 | -0.26 | -0.27 | -0.03 | -0.15 | 1.00 | 0.00 |  |
| Spines (petiole) (SP) | 0.00 | 0.00 | 0.00 | 0.00 | 0.00 | 0.00 | 0.00 | 0.00 | 0.00 | 0.00 | 0.00 | 0.00 | 0.00 | 0.00 | 0.00 | 0.00 | 0.00 | 0.00 | 1.00 |  |
| Number of top teeth (T) | -0.70 | -0.03 | 0.46 | 0.35 | 0.69 | 0.79 | 0.30 | 0.59 | 0.34 | 0.57 | 0.07 | 0.38 | -0.18 | -0.20 | -0.10 | -0.09 | -0.23 | 0.15 | 0.00 |  |

^a^Measured on the alitrunk
