# Supplementary material for: Morphological traits: predictable responses to macrohabitats across a 300 km scale
Source: PeerJ. 2014 Mar 4;2:e271. doi: 10.7717/peerj.271 (PMC3961160; doi:10.7717/peerj.271)
Supplement: Supplementary file 3 [file peerj-02-271-s003.docx]

**Subfamily**	**Morphospecies**	**R**	**P**	**Subfamily**	***Morphospecies***	**R**	**P**	**Subfamily**	**Morphospecies**	**R**	**P**
Amblyoponinae	*Amblyopone sp. 1*	1	0	Formicinae	*Notoncus sp. 1*	3	3	Myrmicinae	*Meranoplus sp. 4*	0	2
Cerapachyinae	*Cerapachys sp. 1*	0	2	Formicinae	*Notoncus sp. 2*	1	0	Myrmicinae	*Meranoplus sp. 5*	1	3
Cerapachyinae	*Cerapachys sp. 2*	1	0	Formicinae	*Notoncus sp. 3*	2	0	Myrmicinae	*Meranoplus sp. 6*	2	0
Cerapachyinae	*Cerapachys sp. 3*	0	1	Formicinae	*Notoncus sp. 4*	3	0	Myrmicinae	*Meranoplus sp. 7*	3	0
Dolichoderinae	*Dolichoderus sp. 1*	2	0	Formicinae	*Nylanderia sp. 1*	3	0	Myrmicinae	*Meranoplus sp. 8*	1	1
Dolichoderinae	*Iridomyrmex sp. 1*	3	3	Formicinae	*Nylanderia sp. 2*	4	2	Myrmicinae	*Meranoplus sp. 9*	1	0
Dolichoderinae	*Iridomyrmex sp. 2*	2	2	Formicinae	*Nylanderia sp. 3*	1	1	Myrmicinae	*Monomorium sp. 1*	3	3
Dolichoderinae	*Iridomyrmex sp. 3*	2	0	Formicinae	*Opisthopsis sp. 1*	1	0	Myrmicinae	*Monomorium sp. 2*	0	2
Dolichoderinae	*Iridomyrmex sp. 4*	0	2	Formicinae	*Plagiolepis sp. 1*	3	0	Myrmicinae	*Monomorium sp. 3*	0	2
Dolichoderinae	*Iridomyrmex sp. 5*	1	1	Formicinae	*Polyrachis sp. 1*	1	0	Myrmicinae	*Monomorium sp. 4*	2	0
Dolichoderinae	*Iridomyrmex sp. 6*	1	2	Formicinae	*Polyrachis sp. 2*	2	0	Myrmicinae	*Monomorium sp. 5*	2	2
Dolichoderinae	*Iridomyrmex sp. 7*	0	1	Formicinae	*Polyrachis sp. 3*	1	0	Myrmicinae	*Monomorium sp. 6*	1	1
Dolichoderinae	*Iridomyrmex sp. 8*	0	1	Formicinae	*Prolasius sp. 1*	1	1	Myrmicinae	*Monomorium sp. 7*	0	2
Dolichoderinae	*Ochetellus sp. 1*	2	1	Formicinae	*Prolasius sp. 2*	1	0	Myrmicinae	*Monomorium sp. 8*	1	0
Dolichoderinae	*Ochetellus sp. 2*	1	0	Formicinae	*Prolasius sp. 3*	2	0	Myrmicinae	*Pheidole sp. 1*	3	3
Dolichoderinae	*Tapinoma sp. 1*	3	3	Formicinae	*Prolasius sp. 4*	2	0	Myrmicinae	*Pheidole sp. 2*	0	1
Dolichoderinae	*Technomyrmex sp. 1*	2	1	Formicinae	*Stigmacros sp. 1*	1	0	Myrmicinae	*Pheidole sp. 3*	1	0
Ectatomminae	*Rhytidoponera sp. 1*	1	1	Formicinae	*Stigmacros sp. 2*	1	1	Myrmicinae	*Pheidole sp. 4*	1	1
Ectatomminae	*Rhytidoponera sp. 2*	3	3	Formicinae	*Stigmacros sp. 3*	1	0	Myrmicinae	*Pheidole sp. 5*	0	6
Ectatomminae	*Rhytidoponera sp. 3*	4	1	Formicinae	*Stigmacros sp. 4*	1	0	Myrmicinae	*Pheidole sp. 6*	4	0
Ectatomminae	*Rhytidoponera sp. 4*	0	3	Heteroponerinae	*Heteroponera sp. 1*	1	1	Myrmicinae	*Pheidole sp. 7*	2	2
Formicinae	*Camponotus sp. 1*	1	2	Myrmeciinae	*Myrmecia sp. 1*	1	0	Myrmicinae	*Pheidole sp. 8*	0	2
Formicinae	*Camponotus sp. 2*	1	0	Myrmeciinae	*Myrmecia sp. 2*	2	0	Myrmicinae	*Pheidole sp. 9*	0	1
Formicinae	*Camponotus sp. 3*	1	2	Myrmeciinae	*Myrmecia sp. 3*	0	2	Myrmicinae	*Pheidole sp. 10*	1	1
Formicinae	*Camponotus sp. 4*	3	1	Myrmeciinae	*Myrmecia sp. 4*	1	0	Myrmicinae	*Pheidole sp. 11*	2	2
Formicinae	*Camponotus sp. 5*	3	0	Myrmeciinae	*Myrmecia sp. 5*	1	0	Myrmicinae	*Pheidole sp. 12*	1	2
Formicinae	*Camponotus sp.6*	1	0	Myrmicinae	*Adlerzia sp. 1*	2	0	Myrmicinae	*Pheidole sp. 13*	3	1
Formicinae	*Camponotus sp. 8*	1	1	Myrmicinae	*Anillomyrma sp. 1*	1	1	Myrmicinae	*Pheidole sp. 14*	4	2
Formicinae	*Camponotus sp. 9*	2	3	Myrmicinae	*Aphaenogaster sp. 1*	2	0	Myrmicinae	*Pheidole sp. 15*	0	3
Formicinae	*Camponotus sp. 10*	0	2	Myrmicinae	*Cardiocondyla sp. 1*	2	2	Myrmicinae	*Pheidole sp. 16*	2	2
Formicinae	*Camponotus sp. 11*	2	0	Myrmicinae	*Cardiocondyla sp. 2*	2	1	Myrmicinae	*Solenopsis sp. 1*	2	4
Formicinae	*Camponotus sp. 12*	0	2	Myrmicinae	*Crematogaster sp. 1*	6	0	Myrmicinae	*Strumigenys sp. 1*	1	0
Formicinae	*Melophorus sp. 1*	1	2	Myrmicinae	*Crematogaster sp. 2*	2	0	Myrmicinae	*Tetramorium sp. 1*	0	2
Formicinae	*Melophorus sp. 2*	0	1	Myrmicinae	*Crematogaster sp. 3*	4	1	Myrmicinae	*Tetramorium sp. 2*	2	2
Formicinae	*Melophorus sp. 3*	0	2	Myrmicinae	*Crematogaster sp. 4*	2	0	Myrmicinae	*Tetramorium sp. 3*	0	2
Formicinae	*Melophorus sp. 4*	0	1	Myrmicinae	*Crematogaster sp. 5*	1	0	Myrmicinae	*Tetramorium sp. 4*	0	3
Formicinae	*Melophorus sp. 5*	1	0	Myrmicinae	*Epopostruma sp. 1*	1	0	Ponerinae	*Hypoponera sp. 1*	0	0
Formicinae	*Melophorus sp. 6*	0	2	Myrmicinae	*Mayriella sp. 1*	1	0	Ponerinae	*Leptogenys sp. 1*	1	1
Formicinae	*Melophorus sp. 7*	1	0	Myrmicinae	*Meranoplus sp. 1*	0	2	Ponerinae	*Pachycondyla sp. 1*	0	3
Formicinae	*Melophorus sp. 8*	2	0	Myrmicinae	*Meranoplus sp. 2*	0	2	Ponerinae	*Pachycondyla sp. 2*	1	0
